# Photodegradation of Flucetosulfuron, a Sulfonylurea-Based Herbicide in the Aqueous Media Is Influenced by Ultraviolet Irradiation

**DOI:** 10.3390/jox11040010

**Published:** 2021-11-06

**Authors:** Arnab Goon, Arijita Bhattacharyya, Bappa Ghosh, Rajiv Rakshit, Anupam Das, Suborna Roy Choudury, Chiranjit Kundu, Pritam Ganguly, Akbar Hossain

**Affiliations:** 1 Department of Chemistry, University of Kalyani, Kalyani, Nadia 741235, West Bengal, India; arnabgoon@gmail.com (A.G.); arijitamam@gmail.com (A.B.); ghosh.bappa3@gmail.com (B.G.); 2 Department of Agricultural Chemicals, Bidhan Chandra Krishi Viswavidyalaya, Mohanpur 741246, West Bengal, India; chirubckv@gmail.com; 3 Department of Soil Science & Agricultural Chemistry, Bihar Agricultural University, Sabour, Bhagalpur 813210, Bihar, India; rajiv.ssaciari@gmail.com (R.R.); anusoil22@gmail.com (A.D.); 4 Department of Agronomy, Bihar Agricultural University, Sabour, Bhagalpur 813210, Bihar, India; subornabau@gmail.com; 5 Bangladesh Wheat and Maize Research Institute, Dinajpur 5200, Bangladesh

**Keywords:** pesticide, photodegradation, sulfonylurea, water, metabolites

## Abstract

Photodegradation (photolysis) causes the breakdown of organic pesticides molecules by direct or indirect solar radiation energy. Flucetosulfuron herbicide often encounters water bodies. For this reason, it is important to know the behavior of the compound under these stressed conditions. In this context, photodegradation of flucetosulfuron, a sulfonylurea-based herbicide, has been assessed in aqueous media in the presence of photocatalyst TiO_2_ and photosensitizers (i.e., H_2_O_2,_ humic acid, and KNO_3_) under the influence of ultraviolet (UV) irradiation. The influence of different water systems was also assessed during the photodegradation study. The photodegradation followed the first-order reaction kinetics in each case. The metabolites after photolysis were isolated in pure form by column chromatographic method and characterized using the different spectral data (i.e., XRD, IR, NMR, UV-VIS, and mass spectrometry). The structures of these metabolites were identified based on the spectral data and the plausible photodegradation pathways of flucetosulfuron were suggested. Based on the findings, photocatalyst TiO_2_ with the presence of ultraviolet irradiation was found effective for the photodegradation of toxic flucetosulfuron residues under aqueous conditions.

## 1. Introduction

Herbicides are chemical substances used to manage weeds in the crop field. Globally, several compounds have been registered based on the requirements and suitability in the application into the field. Earlier, the rate of the herbicide molecules was high and eco-safety was a major concern. However, with recent developments, these are becoming minimized. In India, crops such as rice, wheat, maize, pulses, tea, vegetables, fiber crops etc. are the major recipients of herbicide applications. Compounds such as 2,4-D, pretilachlor, butachlor, Pendimethalin, glyphosate, paraquat etc. are very popular herbicides among Indian farmers since a long time. However, to have better efficacy at lower doses, compounds of ‘fop’ and ‘urea’ groups are coming out as potent replacements.

**Figure 1:**
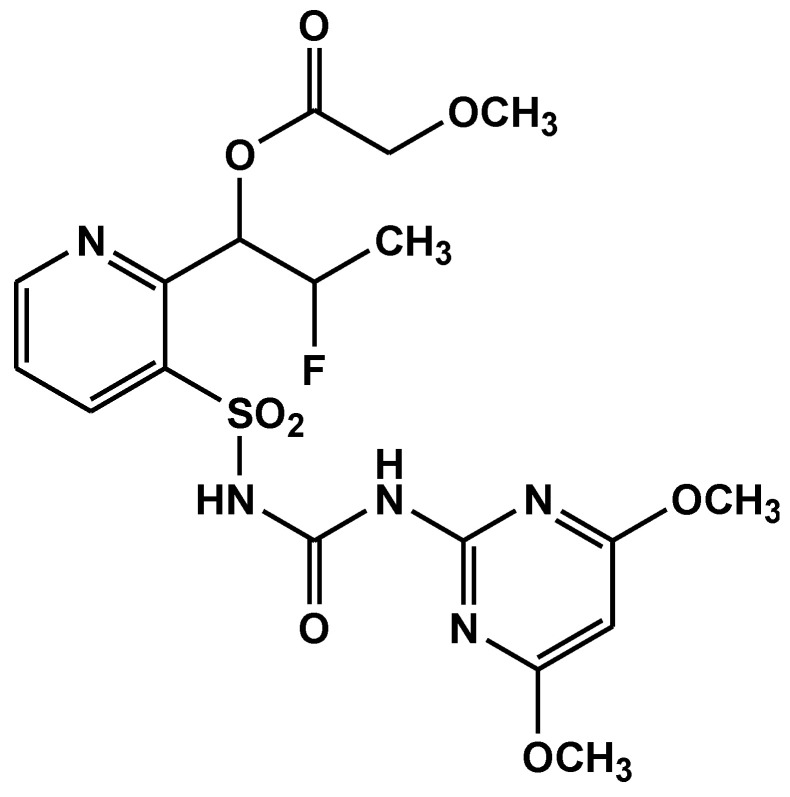
Structure of flucetosulfuron.

Flucetosulfuron (Figure 1) is a sulfonylurea-based newly introduced herbicide (Registered on 27 May 2016) used in rice fields that controls *Echinochloa crusgalli* as well as other important annual and perennial weeds effectively [1,2,3]. Sulfonylurea-based herbicides generally inhibit the acetolactate synthase (ALS) in the biosynthetic pathway of the branch-chain amino acids viz., valine, leucine, and isoleucine. The compound is recently registered in India and farmers have started using it in their respective fields. The application rate of this herbicide is 250 g a.i. ha^−1^ [4]. Thus, the environmental fate of flucetosulfuron in Indian conditions would be an interesting subject. It does not show any toxicity effect even in nursery bed also. Flucetosulfuron is also performing in direct-seeded rice. There are several processes involved such as runoff, leaching, plant uptake, evapotranspiration, degradation (physical, chemical, biological, photo) etc., which ultimately determines the fate of a pesticide compound in the environment [5]. Photodegradation through hydrolysis is one of the major transformation processes affecting the fate of pesticides in the aquatic environment. The reactions of a pesticide with water (hydrolysis) and light (photolysis) are important in predicting its ultimate environmental fate [6].

Photodegradation (photolysis) involves the degradation of organic pesticides under direct or indirect solar radiation. Light energy is usually absorbed either directly by the pesticide molecule, or secondary materials (photocatalyst/photosensitizer) become ‘activated’ by absorbing light energy and then transfer energy to the pesticide molecule. In both cases, pesticide molecules absorb energy, become excited or reactive, and are degraded generally into non-toxic metabolites [6,7]. In photo-generated catalysis, the photocatalytic activity (PCA) depends on the ability of the catalyst to generate free radicals (e.g., hydroxyl radicals: •OH) which can undergo secondary reactions. TiO_2_, H_2_O_2_, KNO_3_ etc. have been exploited to a great extent in pesticides photodegradation [8].

Titanium dioxide (TiO_2_) is a widely known photocatalyst for pesticide degradation in water as it is considered a very efficient catalyst. Unlike other semiconductors, it is non-toxic, stable to photo-corrosion, low cost and suitable to work using sunlight as an energy source [9,10,11]. The mechanism of photodegradation by TiO_2_ occurs through the photo-generated electron-hole pair mechanism. These electron-hole pairs eventually migrate to the interface and produce hydroxyl and superoxide radicals. Hydroxyl and superoxide radicals are the primary oxidizing species in the photo-catalyzed oxidation processes [11].

Nitrate ions (NO_3_^−^) are usually present in the aquatic environment along with nitrite ions (NO_2_^−^), produced by the photolysis of nitrate ions irrespective of geographic nature and agricultural activities [12]. Both the ions can absorb solar radiation and can undergo chemical reactions. Excited nitrite ions produce hydroxyl radicals, nitrogen monoxide, NO_2_ and N_2_O_4_ [13,14]. Thus, the photolysis of nitrite ions in the aquatic environment cannot be therefore neglected.

Advanced oxidation processes (AOP) has been shown capable of degrading pesticides, algal toxins and algal related taste and odor compounds (T&O), endocrine-disrupting compounds (EDCs), pharmaceuticals and personal care products (PPCPs), perfluorinated compounds (PFCs), and many other classes of emerging, recalcitrant compounds [15,16]. AOPs employ strong oxidizing intermediate radical species to degrade pollutants [17]. Most often, the oxidizing radical is the hydroxyl radical (•OH). The advantage of using •OH as an oxidant is the high oxidation potential of •OH which is greater than other strong oxidants, including ozone, hydrogen peroxide, and chlorine dioxide [18].

The role of humic acid (HA) in the aquatic environment has been investigated extensively; numerous studies have depicted that HA sensitizes or mediates the synthesis of reactive intermediates including ^1^O_2_, superoxide anion, and/or H_2_O_2_ in oxygenated water. HA holds varied structures consisting of chromophores that can perform as photosensitizers, either directly from the energized states; or indirectly via ^1^O_2_ [19]. Besides its advantageous role in weed control, there is a concern regarding environmental contamination associated with the use of flucetosulfuron as other chemical herbicides. The compound could potentially enter the surface water by spray drift during application or runoff after application. As this herbicide is used in the flooded rice environment, the determination of the rate and pathways of photolytic degradation in water is vital for defining the environmental impact of flucetosulfuron application. There is information available on the degradation of flucetosulfuron in flooded rice field soils [20,21,22]. However, an extensive study on photodegradation of flucetosulfuron in aqueous media revealing the plausible pathway and metabolites is very limited. It is important to know the behavior of flucetosulfuron under aqueous conditions under the influence of UV irradiation in the presence or absence of photosensitizers. The present investigation was carried out to enumerate the nature of photolytic degradation of flucetosulfuron herbicide in pure water, irrigation water, and river water under the influence of UV irradiation. In this study, the influence of photocatalystTiO_2_ and photosensitizers KNO_3_, H_2_O_2_ and HA on the degradation were examined and characterization of products formed was done to understand their probable mechanism of formation.

## 2. Materials and Methods

### 2.1. Apparatus

The kinetic study along with a characterization of photo metabolite was carried out in Alliance 2695 Separations Module (Waters, Milford, MA, USA) attached with Micromass Quattro Micro triple-quadruple mass spectrometer (Micromass, Manchester, UK) using electrospray ionization in the positive ion (ES+) mode. IR spectra for the characterization of products were analyzed using KBr pellets (1.0 mm) using an infrared spectrophotometer (Make: Perkin Elemer, Model: Spectrum One, Model No: L120-000A). ^1^H NMR spectra were obtained on a JEOL ECS-400 NMR spectrometer using TMS as the internal standard. X-ray crystallographic analysis was done on a Bruker SMART APEXII CCD area-detector diffractometer using graphite monochromatic Mo Kα radiation (λ = 0.71073 Å). X-ray data reduction was carried out using the Bruker SAINT program. The structures were hypothesized by direct methods using the SHELXS-97 program and refinement using SHELXL-97 program. The samples were centrifuged using a high-speed refrigerated centrifuge, Model Avanti J-30I (Beckman coulter, Brea, CA, USA). The rotor head was suitable for holding eight no. of 50 mL (JA-30.50 T1) fluorinated ethylene propylene (FEP) centrifuge tubes (Nalgene, Rochester, NY, USA). Samples were evaporated using a Turbo Vap LV instrument from Caliper Life Science (Hopkinton, MA, USA).

### 2.2. Reagents

Analytical standard of flucetosulfuron (99.8% pure) was procured from Indofil Industries Limited, Mumbai. Analytical grade organic solvents such as acetonitrile (MeCN), ethyl acetate (EA), and hexane were procured from JT Baker (Phillipsburg, NJ, USA). Ammonium acetate (CH_3_COONH_4_), KNO_3_, TiO_2_, H_2_O_2_ and HA were purchased from Merck Life Science Private Limited (Mumbai, India). Acetic acid, silica gel and sodium sulphate (Na_2_SO_4_) were purchased from SRL Pvt. Ltd. (Mumbai).

### 2.3. Experimental Details

#### 2.3.1. Water Samples

The water systems chosen for the study were pure water, irrigation water, and river water. Pure water was collected from Milli-Q (Millipore, Bedford, MA, USA) water purification system. Irrigation water (ground water) was collected from a shallow pump installed in the rice field and river water was collected from the Ganga River. Important quality parameters of the collected water samples were measured and presented in Table 1.

#### 2.3.2. Irradiation Experiment

Flucetosulfuron was irradiated in an aqueous medium under UV light in the presence of photocatalyst and photosensitizers. Aqueous solutions of flucetosulfuron were prepared by dissolving 10 mg of flucetosulfuron in 1 L pure water separately. To understand the effect of different photocatalyst/photosensitizers on photodegradationTiO_2_, KNO_3_, H_2_O_2_ and HA were mixed (50 mg L^−1^) separately and irradiated. The irradiation was done by UV light (λ _max_ ≥ 250 nm), and the inside reactor was fitted with a high-pressure mercury lamp (125 Watt, HPK, Philips) encapsulated with a water-cooled pyrex filter to maintain a constant solution temperature (25 °C) with continuous stirring by a magnetic stirrer. The flasks were firmly covered with aluminum foil to prevent any kind of contamination or other exposures. Samples were collected at intervals of 0, 2, 6, 12, 24, 36, 48, 60, 72, 84, 96, 108, and 120 h from the irradiated solution for further analysis. Control samples without photocatalyst or photosensitizer for each water system have been processed in the same manner to find out the actual effects of these additives.

### 2.4. Sample Extraction

Each water sample (10 mL) was taken in a 50 mL centrifuge tube and 10 mL ethyl acetate was added to it. To this mixture 100 µL acetic acid was added and shaken for 5 min with the help of vortex. Afterwards, the sample was centrifuged at 10,000 rpm for 10 min. From it, 2 mL supernatant ethyl acetate fraction was collected and evaporated to dryness via nitrogen evaporator. Volume was reconstituted with 2 mL acetonitrile and filtered through 0.2 µm membrane filter. The samples were then analyzed in LC–MS/MS.

### 2.5. LC–MS/MS Analysis

Flucetosulfuron residues were analyzed in liquid chromatography–tandem mass spectrometry. The mobile phase constituted with 5% mobile phase A [acetonitrile/water 90/10 (*v*/*v*) with 5 mM ammonium acetate] and 95% mobile phase B [acetonitrile/water 10/90 (*v*/*v*) with 5 mM ammonium acetate]. The HPLC separation was performed by injecting 20 µL sample via auto sampler on a Symmetry C_18_ (5 µm; 2.1 × 100 mm) column (Waters, Milford, MA, USA). The solvent flow rate was 0.3 mL min^−1^ and the total run time was 5 min. The compounds along with their retention times (RTs), quantifier ions, and qualifier ions are presented in Table 2. The optimized MS instrument parameterized with capillary voltage, 1.00 kV; cone voltage, 32 V; source temperature, 120 °C; desolvation temperature, 350 °C; desolvation gas flow, 650 L h^−1^ nitrogen; cone gas flow, 50 L h^−1^; argon collision gas pressure to 3.5 × 10^−3^ psi for MS/MS. The analysis of flucetosulfuron was performed by multiple reaction monitoring (MRM) with three mass transitions and dwell time 0.150 s.

### 2.6. Extraction, Isolation, and Identification of Products from Solution

For isolation of products, flucetosulfuron was irradiated in eight different sets, each containing 250 mg of the compound dissolved in 1 L pure water containing TiO_2_ as photocatalyst. The solution mixtures were irradiated by UV light for 12 h with continuous stirring by a magnetic stirrer. The irradiated solvent mixtures were extracted with ethyl acetate. The combined crude extract was then subjected to column chromatography over silica gel (100–200 mesh) and the column was eluted with solvents of increasing polarity (hexane to ethyl acetate, in an increasing ratio) to isolate the photolytic products in pure form. Isolation of different eluted products was confirmed with the help of thin-layer chromatography. After isolation, the probable products were subjected to X-ray diffraction (XRD) study, nuclear magnetic resonance (NMR) analysis, IR analysis, and mass spectrometric study for identification and confirmation of the structures.

### 2.7. Method Validation Parameters

Performance of the analytical method had been evaluated based on linearity, accuracy, precision and sensitivity [23]. Flucetosulfuron standard had been injected as 0.01, 0.02, 0.03, 0.05 and 0.10 mg L^−1^ to work out the calibration curve. Each type of water was spiked with the compound at the level of 0.03, 0.15 and 0.30 mg L^−1^ to judge the accuracy of the method. Precision (intra-laboratory) of the method was estimated based on the Horwitz ratio (HorRat) which may be expressed as HorRat = RSD/PRSD [24,25]. Here, RSD represents relative standard deviation and PRSD (predictive RSD) is 2C^–0.15^ of which C stands for concentration in ppb level. The sensitivity of the method was evaluated based on the limit of detection (LOD) and limit of quantification (LOQ). LOD and LOQ were set at signal:noise ratio of 3:1 and 10:1, respectively.

### 2.8. Data Analysis

Dissipation kinetics of flucetosulfuron followed first-order kinetics in each water system as the initial amount of the herbicide degraded with an increment of time. This can be mentioned as: *C_t_ = C_0_e^−kt^* where *C*_0_ stands for initial concentration; *C*_t_ is the amount of pesticide residue at time *t* and *k* is the dissipation rate constant calculated in hours. By taking the logarithm (ln) in each side of the equation, this can be re-written as ln *C_t_* = ln *C*_0_ − *kt*. The half-life (*t*_1/2_) value of flucetosulfuron can be calculated as: *t*_1/2_ = ln 2/*k* [26].

## 3. Results and Discussion

### 3.1. Method Validation

**Figure 2:**
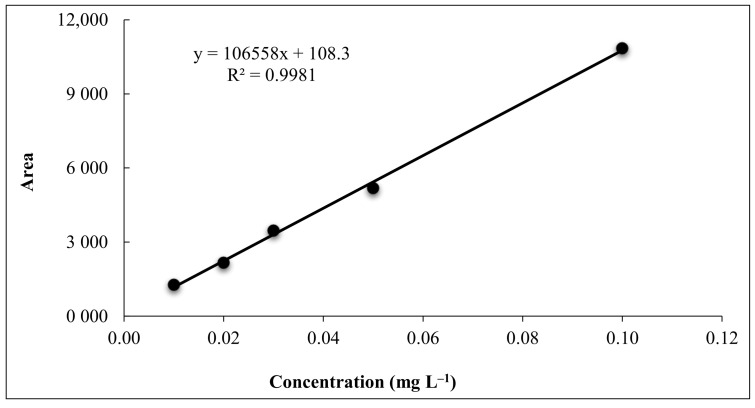
Calibration curve of flucetosulfuron.

Different method validation parameters have been presented in Table 3. The analytical method was found linear in the said range as the correlation coefficient (R^2^) of the calibration curve was 0.998 (Figure 2). The average recovery was ranged between 83.33–92.83% irrespective of substrate and level of spiking which showed acceptable accuracy. The method was found precise as the HorRat values were observed between the acceptable ranges of 0.5 to 2.0. LOD and LOQ for flucetosulfuron were found 0.01 and 0.03 µg mL^−1^, respectively. Based on these parameters, the performance of the analytical method was found quite satisfactory.

### 3.2. Photodegradation Kinetics

Results regarding the dissipation of flucetosulfuron in different waters samples have been presented in Table 4. Following the dissipation kinetics of flucetosulfuron in different systems, it was found that the half-life (*t*_1/2_) of the compound was highest in pure water without photocatalyst/photosensitizer. Photodegradation of flucetosulfuron was rapid in the presence of photocatalyst TiO_2_ than photosensitizers irrespective of water systems. The half-life of flucetosulfuron was 30.54–55.45 h in the presence of TiO_2_, whereas KNO_3_, H_2_O_2_, and HA showed about 1.2–2.0 times higher half-life than TiO_2_ mediated photocatalysis. A similar observation was found when sulfonylurea herbicides other than flucetosulfuron were degraded in the presence of TiO_2_ [27,28]. Other additives i.e., KNO_3_, H_2_O_2_ and HA also facilitated quick photodegradation of the herbicide than the system without them.

### 3.3. Column Chromatographic Isolation and Characterization of Metabolites

The photodegradation study revealed that the half-life of flucetosulfuron was lowest in pure water with TiO_2_ as photocatalyst under UV irradiation. It was recorded that flucetosulfuron was degraded 58.39% after 12 h of UV irradiation. Thus, it can be expected that the yield of metabolites will be high after 12 h of UV irradiation. After the stated time interval, the system was taken for extraction. The extracted fraction was concentrated, and the crude concentrate was subjected to column chromatography over silica gel. The column was eluted with non-polar solvent and thereafter increased the polarity of eluting solvent stepwise. The elution scheme and the relative amount of the metabolites of flucetosulfuron are presented in Table 5.

**Figure 3:**
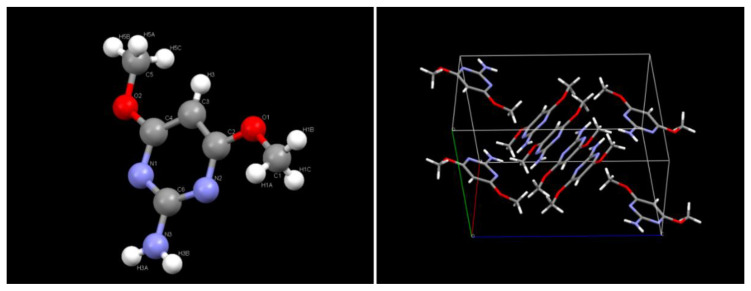
X-ray Crystal structure of product M1 and packing of M1 within a unit crystal cell.

**Figure 4:**
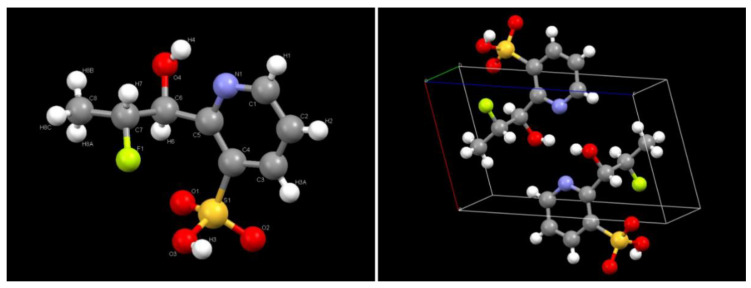
X-ray Crystal structure of product M2 and packing of M2 within a unit crystal cell.

The first isolated compound M_1_ was eluted with solvent composition, hexane—ethyl acetate (95:5) with a single spot in TLC. The compound M_1_ was a colorless crystal. For identification of the crystal M_1_, it was subjected to X-ray diffraction (XRD) study. Selected crystal data and data collection parameters for the compound M_1_ were given in Table 6. The three-dimensional crystal structure of the product M_1_ recorded from XRD analysis is shown in Figure 3. The X-ray crystallographic data conclusively identifies the product M_1_ as 4,6-dimethoxy-pyrimidin-2-ylamine. This metabolite was formed via cleavage of the C-N linkage adjacent to pyrimidine moiety. The compound is also being found during flucetosulfuron metabolism in artificial gastrointestinal juice [29]. This phenomenon has also been reported in the metabolism of similar compounds such as sulfosulfuron [30], imazosulfuron [31] and rimsulfuron [32]. The second isolated compound M_2_ was eluted with solvent composition, hexane–ethyl acetate (70:30) with a single spot in TLC. The compound M_2_ was a colorless crystal. For identification of the crystal M_2_, it was subjected to X-ray diffraction (XRD) study. X-ray crystallographic study of M_2_ was done by the same instrument as described in the previous study. Selected crystal data and data collection parameters for the compound M_2_ were given in Table 6. The three-dimensional crystal structure of the product M_2_ is shown in Figure 4. The above X-ray crystallographic data conclusively identifies isolated product M_2_ as 2-(2-fluoro-1-hydroxy-propyl)-pyridine-3-sulfonic acid.

The third isolated compound M_3_ was eluted with solvent composition, hexane–ethyl acetate (50:50) with a single spot in TLC. Compound M_3_ was found at a very low amount. The full scan ESI–MS analysis of M_3_ showed a mass spectrum of *m/z* 415.17 (M_3_ + H) and *m/z* 437.16 (M_3_ + Na). The identity of M_3_ was not fully confirmed, but the ESI-MS analysis and considering known (5) metabolic pathways of the same compound strongly support M_3_ as an important intermediate *N*-((4,6-dimethoxypyrimidin-2-yl) amino carbonyl)-2-(1-hydroxy-2-fluoropropyl)-3-pyridine sulfonamide. This particular metabolite is also reported to be formed during the in-vitro metabolism of flucetosulfuron in artificial gastrointestinal juice [29] as well as in rice plant and barnyard grass [33]. The probable formation of this metabolite is also demonstrated during in-vitro metabolism of the same herbicide by human liver microsome [34]. Moreover, artificial gastrointestinal juices caused rapid degradation of flucetosulfuron hindered its translocation to blood during any accidental oral intake [34]. It implies that the possibility of its human toxicity is quite negligible.

**Figure 5:**
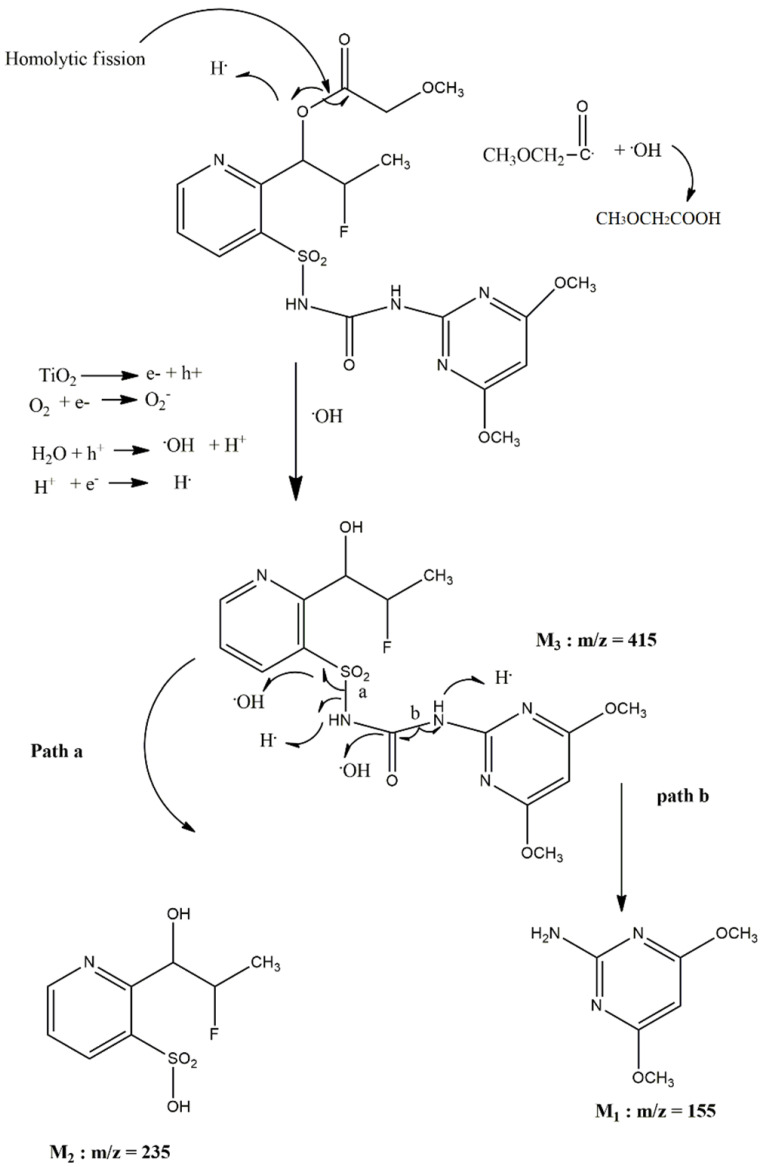
Plausible pathway of photodegradation of flucetosulfuron.

The fourth isolated compound M_4_ was a white solid eluted by solvent mixture hexane–ethyl acetate (10:90). Structure elucidation of M_4_ was done with the help of infrared (IR) spectroscopy, proton nuclear magnetic resonance (NMR) spectroscopy and mass spectrometry. Full scan ESI-MS analysis of compound M_4_ showed the mass spectrum with a base peak at *m*/*z* 488. The IR spectrum of compound M_4_ showed several absorption bands. The respective probable groups found in the spectrum were,(C=O) stretching frequency for carbonyl group (1719.63 cm^−1^); C=O stretching frequency for ester group (1768.78 cm^−1^); S=O stretching frequency for O=S=O (SO_2_) group (1366.82 cm^−1^); aromatic C=C stretching and N-H deformation frequency for amide (1636.25 cm^−1^); aromatic C=C stretching (1453.86 and 1582.12 cm^−1^) and N-H stretching band for secondary amide (3191.18 cm^−1^). The ^1^H NMR spectrum showed the characteristic signals at δ1.37–1.39 (d, 3H), 3.13 (s, 3H), 3.92 (s, 6H), 4.05 (s, 2H), 5.99 (d, 1H), 6.71–6.75 (m, 1H), 7.89 (s, 1H), 8.26–8.29 (dd, 1H), 8.76–8.77 (dd, 1H), 8.88–8.90 (dd, 1H), 10.66 (s, 1H) and 13.26 (s, 1H). This ^1^H NMR spectrum confirms M_4_ as unreacted parent compound flucetosulfuron. The plausible mechanistic pathway of the photodegradation of flucetosulfuron is presented in Figure 5 which is supported by the experimental evidence cited in the literature [35,36]. All these M_1_, M_2_, and M_3_ metabolites are found to be formed when flucetosulfuron is undergone both acid and alkaline hydrolysis. It was reported that ester hydroxylation, which refers to the hydrolysis reaction of the ester bond, was the initial step followed by sulfonylurea bridge cleavage [37].

## 4. Conclusions

The present study deals with an important aspect of flucetosulfuron photodegradation in water. Herbicides are used to contaminate water reservoirs very often through different movements such as surface runoff, seepage, leaching, industry effluents etc. It is obvious to find out the dissipation kinetics and the fate of the herbicide. The behavior under the influence of different photocatalyst or photosensitizers has been observed, in which photocatalyst TiO_2_ played a significant role compared to others. Three metabolites could be isolated after photodegradation of flucetosulfuron. Out of the isolated metabolites, the structure of two was confirmed as 4, 6-dimethoxy-pyrimidin-2-ylamineand 2-(2-fluoro-1-hydroxy-propyl)-pyridine-3-sulfonic acid. The identity of the third isolate was not fully confirmed; however, the MS spectral information and known metabolic pathways suggest it as an important intermediate *N*-((4,6-dimethoxypyrimidin-2-yl) aminocarbonyl)-2-(1-hydroxy-2-fluoropropyl)-3-pyridinesulfonamide. The current investigation provided us with the complete transformation process of flucetosulfuron under the influence of TiO_2_ —UV system. This leads to the future endeavor where TiO_2_—UV system could be used as a viable decontamination option for flucetosulfuron residues present in water. However, a complete toxicity study needs to be performed in support of this decontamination process, although rapid degradation of flucetosulfuron by artificial gastrointestinal juices in-vitro metabolism indicates its negligible human toxicity.

## Figures and Tables

**Table 1:** Water quality parameters.

Parameters	Pure Water	Irrigation Water	River Water
pH	7.00	6.67	7.48
Electric Conductivity (EC) (dS m^−1^)	ND	0.47	0.35
Dissolved Oxygen (DO) (mg L^−1^)	5.60	6.80	7.30
Biological Oxygen Demand (BOD) (mg L^−1^)	ND	1.20	1.80
Chemical Oxygen Demand (COD) (mg L^−1^)	4.00	24.00	44.00
Total Solid (TS) (mg L^−1^)	ND	41.00	340.00
Total Dissolved Solid (TDS) (mg L^−1^)	ND	24.90	252.00
Total Soluble Solid (TSS) (mg L^−1^)	ND	16.10	88.00
Total Hardness (mg CaCO_3_ L^−1^)	ND	220.00	156.00

ND-Not detected.

**Table 2:** Overview of the LC–MS/MS analysis of the flucetosulfuron.

Pesticide	RT (min)	Q	Q_1_	CV (V)	CE (V)	Q_2_	CV (V)	CE (V)
Flucetosulfuron	0.69	487.87	155.89	32	16	273.00	32	28

RT: retention time; Q: protonated parent ion; Q_1_: quantifier ion; Q_2_: second transition; CV: cone voltage; CE: collision energy.

**Table 3:** Results of method validation of flucetosulfuron.

Substrate	Spiked Level (mg L^−1^)	Parameters					
		Mean Residue (mg L^−1^)	SD	RE (%)	RSD (%)	PRSD	HorRat
Pure water	0.03	0.03	0.00	85.00	11.28	9.77	1.15
	0.15	0.13	0.01	87.44	9.80	7.64	1.28
	0.30	0.28	0.03	92.83	11.19	6.83	1.64
Irrigation water	0.03	0.03	0.00	83.33	13.09	9.80	1.34
	0.15	0.14	0.01	92.11	6.61	7.59	0.87
	0.30	0.28	0.03	92.67	9.49	6.83	1.39
River water	0.03	0.03	0.00	86.67	8.27	9.75	0.85
	0.15	0.14	0.01	92.67	9.11	7.58	1.20
	0.30	0.27	0.03	90.67	10.38	6.85	1.51

**Table 4:** Dissipation of flucetosulfuron in water under UV irradiation.

Time (h)	Dissipation (%)														
	Control			TiO_2_			KNO_3_			H_2_O_2_			HA		
	PW	IW	RW	PW	IW	RW	PW	IW	RW	PW	IW	RW	PW	IW	RW
0	0.00	0.00	0.00	0.00	0.00	0.00	0.00	0.00	0.00	0.00	0.00	0.00	0.00	0.00	0.00
2	1.99	7.71	1.47	20.91	10.99	5.21	12.85	8.35	2.43	15.41	7.42	8.02	10.13	12.52	10.09
6	12.35	10.16	11.15	39.13	25.37	24.00	24.19	12.80	10.70	33.61	22.99	16.51	21.29	21.37	18.21
12	25.93	16.99	19.98	58.39	40.48	40.55	53.64	23.93	18.68	40.45	30.68	32.17	28.61	30.79	22.16
24	34.76	31.74	28.81	75.49	62.00	58.22	62.96	41.93	38.13	52.91	43.22	44.43	48.87	41.90	39.96
36	40.55	37.01	38.28	87.54	75.37	71.20	76.32	60.76	56.71	62.41	56.04	55.28	50.28	55.27	43.91
48	43.87	43.16	47.42	92.28	83.52	81.41	84.41	70.13	71.11	77.32	68.41	58.49	62.76	68.46	52.55
60	49.10	46.97	52.89	BDL	89.10	86.62	87.15	80.98	79.18	83.96	77.93	65.57	66.14	70.81	61.29
72	52.71	50.39	56.78	BDL	91.03	90.50	92.21	89.15	83.17	85.80	79.21	74.91	79.36	73.16	71.38
84	57.93	56.15	60.15	BDL	95.05	91.22	BDL	91.09	84.92	91.22	81.59	79.15	83.96	79.47	75.65
96	62.58	59.38	63.51	BDL	BDL	93.05	BDL	94.25	91.54	96.22	84.98	82.55	85.83	82.30	78.56
108	64.67	67.97	66.67	BDL	BDL	BDL	BDL	BDL	92.22	BDL	90.38	86.79	92.96	85.31	80.75
120	66.57	70.61	73.82	BDL	BDL	BDL	BDL	BDL	BDL	BDL	BDL	90.75	BDL	88.23	86.78
Regression Equation	y = 3.9730 − 0.0038x	y = 3.9804 − 0.0041x	y = 3.9495 − 0.0045x	y = 3.9270 − 0.0227x	y = 4.0025 − 0.0150x	y = 3.9340 − 0.0125x	y = 3.9358 − 0.0148x	y = 4.0628 − 0.0129x	y = 4.0262 − 0.0108x	y = 3.9611 − 0.0129x	y = 3.9969 − 0.0088x	y = 3.9946 − 0.0079x	y = 4.0166 − 0.0094x	y = 3.9648 − 0.0074x	y = 3.9573 − 0.0068x
Half-life (T_1/2_) (h)	182.41	169.06	154.30	30.54	46.21	55.45	46.83	53.73	64.18	53.73	78.77	87.74	73.74	93.67	101.93

Below Detectable Limit (BDL) = <0.030 mg L^−1^; PW—Pure water, IW—Irrigation water, RW—River water.

**Table 5:** Extraction scheme and the relative number of metabolites.

Products	Fraction	Relative Amount (%)
M_1_	Hexane: Ethyl Acetate (95:5)	17.75
M_2_	Hexane: Ethyl Acetate (70:30)	10.15
M_3_	Hexane: Ethyl Acetate (50:50)	1.0
Flucetosulfuron (M_4_)	Hexane: Ethyl Acetate (10:90)	36.60
Unidentified	-	34.50

**Table 6:** Crystallographic data for products M_1_ and M_2_.

Parameters	Product M_1_	Product M_2_
Empirical Formula	C_6_H_9_N_3_O_2_	C_8_H_10_FNO_4_S
Formula Weight	155.16	235.24
space group	Monoclinic, C2/c	Triclinic, P-1
*a*, Å	12.51 (2)	7.5034 (14)
*b*, Å	8.50 (2)	8.0341 (16)
*c*, Å	14.69 (3)	10.394 (3)
*α,* deg	90.00	67.842 (5)
*β,* deg	105.98 (6)	94.670 (2)
*γ,* deg	90.00	63.489 (3)
*V*, Å^3^	1501 (6)	505.45 (19)
*Z*	8	2
Crystalsize, mm^3^	0.14 × 0.08 × 0.04	0.48 × 0.32 × 0.21
Color	Colorless	Colorless
*T*, K	273 (2)	273 (2)
μ, mm^−1^	1.372	1.546
Absorption correction method	Multi-scan	Multi-scan
T_min_/T_max_	0.991/0.996	0.881/0.933
Data/parameters	1264/102	1819/139
θ Range (°)	2.89–25.21	2.17–25.26
Δ*ρ*_max_, Δ*ρ*_min_	0.236, −0.331	0.452, −0.419
Final R indices [F^2^ > 2σ(F^2^)]	*R*_1_ = 0.0713 *wR*_2_ = 0.1770	*R*_1_ = 0.0402 *wR*_2_ = 0.1064
Final R indices (all data)	*R*_1_ = 0.1450 *wR*_2_ = 0.2279	*R*_1_ = 0.0478 *wR*_2_ = 0.1122
GOF	0.996	1.052

## Data Availability

Not applicable.

## Ethical Statement

No living organism (human or animal) was involved in conducting the present experiments.

## Abbreviations

ALS	Acetolactate synthase
AOP	Advanced Oxidation processes
CE	Collision energy
CH_3_COONH_4_	Ammonium acetate
CV	Cone voltage
EA	Ethyl acetate
EDC	Endocrine-disrupting compound
ESI-MS	Electrospray ionization-Mass spectroscopy
FEP	Fluorinated ethylene propylene
H_2_O_2_	Hydrogen peroxide
HA	Humic acid
HPLC	High-pressure liquid chromatography
KNO_3_	Potassium nitrite
kV	kilo volt
LC–MS/MS	Liquid chromatography-Mass spectroscopy/Mass spectroscopy
LOD	limit of detection
LOQ	limit of quantification
MeCN	Acetonitrile
MRM	Multiple reaction monitoring
^•^NO_2_	nitrite ions produce hydroxyl radicals
N_2_O_4_	nitrogen monoxide
Na_2_SO_4_	Sodium sulphate
NMR	Nuclear magnetic resonance
•OH	hydroxyl radicals
^1^O_2_	Singlet oxygen
PCA	Photocatalytic activity
PFC	Perfluorinatedcompound
PPCP	Pharmaceuticals and personal care product
RT	Retention times
T&O	Taste and odor compounds
TiO_2_	Titanium oxide
UV	Ultraviolet
XRD	X-ray diffraction
